# Evaluating Retinal and Choroidal Perfusion Changes After Ocular Massage of Healthy Eyes Using Optical Coherence Tomography Angiography

**DOI:** 10.3390/medicina56120645

**Published:** 2020-11-26

**Authors:** Felix Rommel, Sabine Lüken, Michelle Prasuhn, Maximilian Kurz, Vinodh Kakkassery, Salvatore Grisanti, Mahdy Ranjbar

**Affiliations:** Department of Ophthalmology, University of Lübeck, Ratzeburger Allee 160, 23538 Lübeck, Germany; sabine.lueken@uksh.de (S.L.); michelle.prasuhn@uksh.de (M.P.); maximilian.kurz@uksh.de (M.K.); vinodh.kakkassery@uksh.de (V.K.); Salvatore.Grisanti@uksh.de (S.G.); eye.research101@gmail.com (M.R.)

**Keywords:** OCTA, ocular massage, retinal perfusion, choroidal perfusion, choriocapillaris, Sattler’s layer, Haller’s layer, intraocular pressure

## Abstract

*Background and objectives:* Ocular massage (OM) is used as a treatment option for acute retinal artery occlusion, under the assumption that it induces vessel dilatation and enhances perfusion. Since evidence of ocular perfusion alteration due to OM is lacking, we investigate the impact of OM on the hemodynamics of the posterior pole in healthy eyes in a noninvasive fashion by using optical coherence tomography angiography (OCTA). *Materials and Methods:* A prospective study was conducted on healthy volunteers, each of whom underwent measurements of intraocular pressure (IOP), central macular thickness (CMT), subfoveal choroidal thickness (SFCT), radial peripapillary capillary perfusion (RPCP), superficial capillary plexus perfusion (SCPP), deep capillary plexus perfusion (DCPP), choriocapillaris perfusion (CCP), Sattler’s layer perfusion (SLP) and Haller’s layer perfusion (HLP) before and after OM. OM was performed for 2 min, consisting of 10-s turns of compression and decompression of the globe. *Results:* A total of 21 eyes from 21 participants (median age 29) were included. After OM, IOP significantly declined (*p* < 0.001), while SFCT (*p* < 0.005), SCPP (*p* < 0.001), DCPP (*p* = 0.004) and CCP (*p* = 0.008) significantly increased. CMT, RPCP, SLP and HLP did not show any significant alteration due to OM. Changes in SCPP correlated positively with changes in CCP and vice versa. *Conclusions:* OCTA-based analysis in healthy adults following OM demonstrated a significant increase of retinal perfusion values, assumed to be due to failure of autoregulatory mechanisms. These findings may indicate a positive effect of OM as a treatment option for patients with acute retinal artery occlusion.

## 1. Introduction

Ocular perfusion mainly depends on the difference between the local arterial blood pressure (BP) and the intraocular pressure (IOP). However, several investigations have revealed that moderate changes in IOP or BP did not significantly affect the retinal blood flow, in contrast to the choroidal vasculature [1,2,3,4,5]. Responsible for this insensitivity to changes in systemic perfusion pressure within the retinal vasculature is the absence of a neuronal innervation. Therefore the retinal perfusion is mainly under autoregulation by myogenic and local metabolic mechanisms, leading to a constant blood flow during ocular perfusion pressure changes within a certain range [6,7].

It has been demonstrated that IOP can be significantly affected by any contact that touches or indents the ocular surface [8,9,10]. Therefore, maneuvers such as ocular massage (OM) or habitual eye rubbing could influence the retinal and choroidal perfusion. OM in turns of compression and decompression is still a treatment option for acute occlusions of retinal arteries, with the intention of inducing IOP fluctuations and retinal arterial dilatation, although evidence is lacking [11,12,13,14]. The development of optical coherence tomography angiography (OCTA) enables the assessment of the retinal and choroidal vascular network in vivo and in real time by creating slab-segmented angiograms in a noninvasive fashion [15,16,17].

In the present study, we take advantage of this noninvasive imaging technique to investigate structural and functional vascular alterations of the retina and the choroidal sublayers in healthy eyes after undergoing OM.

## 2. Methods

**Figure 1:**
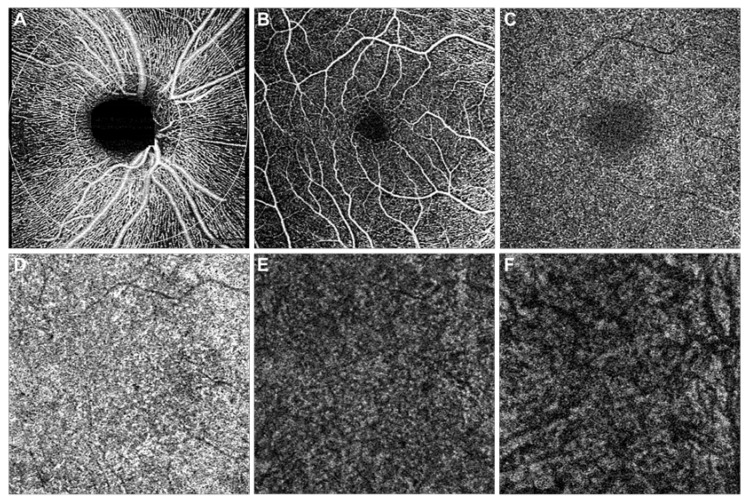
OCTA imaging of the posterior pole in a healthy participant. En face angiogram of radial peripapillary capillaries (**A**), superficial capillary plexus (**B**), deep capillary plexus (**C**), choriocapillaris (**D**), Sattler’s layer (**E**) and Haller’s layer (**F**).

Participants for this prospective observational study were enrolled from the medical staff of the Department of Ophthalmology at the University of Lübeck between August 2019 and January 2020. The study was approved by the institutional review board (ethical code number 19-347, approved on 30 July 2019) and was conducted in accordance with the Declaration of Helsinki. Written informed consent was obtained individually before enrolment. Exclusion criteria were: (1) any history of ocular disease or ocular surgery; (2) evidence or history of systemic disorders, including cardiovascular disease, antihypertensive drug use and diabetes mellitus. At baseline, all included participants underwent an examination including BP, refraction, best-corrected visual acuity (BCVA) in Snellen, axial length (AL) measurement, slit-lamp biomicroscopy and IOP. IOP measurements were obtained using the TONOREF III (NIDEK Co., Ltd., Gamagori, Japan) noncontact tonometer. Imaging was performed on all subjects without prior pupil dilatation using the Zeiss Cirrus HD-OCT (AngioPlex, CIRRUS HD-OCT model 5000, Carl Zeiss Meditec, Inc., Dublin, CA, USA) by a single, trained operator (F.R.). Each imaging session included Enhanced Depth Imaging (EDI)-OCT scans (10 × 10 mm²) and optical coherence tomography angiography (OCTA) volumetric scans (6 × 6 mm²) of the posterior pole, as well as OCTA scans of the radial peripapillary capillaries (RPC). OCTA scans with a signal strength ≥7 and without motion, segmentation or projection artifacts were utilized to avoid misinterpretation [18,19]. Total macular volume (TMV), central macular thickness (CMT) and RPC perfusion (RPCP) were automatically acquired by the device (Figure 1A). Subfoveal choroidal thickness (SFCT) was measured perpendicularly below the fovea by two experienced graders (F.R. and M.P.) and the obtained values were averaged for further analysis. OCTA images were automatically segmented in all B-scans according to the manufacturer’s default setting to produce en face images of the superficial capillary plexus (SCP) and the deep capillary plexus (DCP) (Figure 1B,C). Manual segmentation (F.R.) was performed to get 20 µm slabs of the choriocapillaris (CC), Sattler’s layer (SL) and Haller’s layer according to previously published protocols [3,17,19,20,21] (Figure 1D–F).

Each acquired en face image was exported into ImageJ (NIH, Version 1.52e, Bethesda, Rockville, MD, USA) and binarized using the Otsu method, an automatic threshold selection from gray-level histograms, by using the command path Image > Adjust > Threshold > Auto in order to determine the percentage of white and black pixels [22]. As in previous publications, SCP perfusion (SCPP), DCP perfusion (DCPP) and CC perfusion (CCP) were calculated by recording the percentage of white pixels, while for SL perfusion (SLP) and HL perfusion (HLP) black pixels were scored [4,23,24].

After baseline examination, participants were asked to perform OM simultaneously on both eyes. Gentle pressure was applied through the closed eyelids to the globe with the index finger and middle finger for 10 s, followed by a 10 s release. This procedure was performed for a period of 2 min. Immediately after the OM, participants underwent another examination of both eyes including noncontact IOP, EDI-OCT and OCTA. The follow-up examination had to be done within 3 min after completed OM. For further analysis only one eye of each participant was included and laterality was assigned by chance, resulting in 12 of the studied eyes being right and 9 being left.

Statistical calculations were performed using IBM SPSS (Version 24.0, Chicago, IL, USA). Power calculation was performed using G*Power (Version 3.1, Düsseldorf, Germany) and a sample size of 19 was calculated to achieve a power of at least >90%. BCVA in decimal Snellen was converted to the logarithm of the minimum angle of resolution (logMAR). Mean arterial pressure (MAP) was calculated based on the formula 2/3 diastolic BP + 1/3 systolic BP. The Shapiro–Wilk test was used to check the normality of the obtained data. As the data were found to be distributed non-normally, variables were summarized in terms of median and range. The nonparametric Wilcoxon signed-rank test was used to compare baseline and follow-up values of the same eye. Possible interaction between the morphological and functional retinal parameters was analyzed using Spearman’s correlation analysis. A value of *p* < 0.05 was considered statistically significant.

## 3. Results

A total of 21 eyes from 21 healthy participants enrolled in this study were included in the analysis. Demographic and clinical data are reported in Table 1. Eleven (52.4%) female and ten (47.6%) male participants were included in this study, with a median age of 29 years.

Changes in IOP as well as anatomical and functional retinal values at baseline and after OM are reported in Table 2. The follow-up examination revealed a statistically significant IOP drop from 16 mmHg to 14 mmHg (*p* < 0.001) after OM. While CMT (*p* = 0.114) and TMV (*p* = 0.323) did not change, SFCT increased significantly from 342.7 µm to 367 µm (*p* = 0.005). The RPCP did not change significantly after OM (*p* = 0.407), while both the SCPP and DCPP significantly increased from 27.7% to 30.4% (*p* < 0.001) and from 38.6% to 38.7% (*p* = 0.004), respectively. Likewise, CCP rose from 47.4% to 48.2% (*p* = 0.008), while SLP (*p* = 0.304) and HLP (*p* = 0.554) did not change significantly.

Differences in IOP and perfusion metrics due to OM are correlated in Table 3. Changes in IOP did not show any significant correlation with changes of retinal or choroidal perfusion. Statistically significant positive correlation was observed between SCPP and CCP (r = 0.704; *p* <0.001). Furthermore, changes in SCPP negatively correlated with changes in SLP (r = −0.518; *p* = 0.016). Statistically significant negative correlation was also found between changes in CCP and HLP (r = −0.56; *p* = 0.008) and positive correlation between SLP and HLP (r = 0.703; *p* = <0.001).

## 4. Discussion

In this prospective OCTA-based study, we investigated retinal and choroidal vascular alterations in healthy eyes after 2 min of OM with alternating 10-s turns of compression and decompression. To the best of our knowledge, this is the first study demonstrating statistically significant increases of SCPP, DCPP and CCP after digital massage of the globe. Furthermore, we were able to demonstrate a significant thickening of SFCT, while SLP and HLP stayed steady.

OM is believed to be a simple procedure to temporary reduce IOP and induce retinal arterial vasodilatation [11]. Furthermore, OM improves the aqueous flow through the surgical site thus enlarging the filtration bleb after trabeculectomy and might be useful for focal vitreomacular traction release [25,26]. The present study was able to demonstrate a significant drop of 2 mmHg (mean: 2.71 mmHg) in IOP after performing OM. These results are in line with reported IOP drops of 2.4 mmHg and 3.73 mmHg immediately after ocular rubbing by Mansour et al. and Lam et al., respectively [27,28]. Lower IOP after OM is thought to occur due to accelerated aqueous outflow via the trabecular meshwork and reduced vitreous volume [29].

Due to its potential to lower IOP through increasing retinal artery perfusion pressure and possibly mechanically facilitate the disintegration of a thrombus, OM is used as an acute treatment option for retinal artery occlusion. However, it is unclear to what extent the compression and decompression of OM can give rise to retinal capillary blood flow. In this study, we were able to demonstrate a statistically significant improved perfusion of the central SCP as well as the DCP following OM by using OCTA. However, RPCP stayed steady after performing OM. One explanation is the fact that the retinal blood flow is mainly determined by autoregulatory mechanisms and local factors with insensitivity to systemic perfusion changes and changes in IOP [4,30,31]. The mechanism responsible for autoregulation within the retinal circulation seems to be the absence of neuronal innervation in retinal vascular beds in contrast to the choroid. Histological studies have shown a rich supply of autonomic innervation for the choroid but the nerves do not go further into the retina [32]. Hence, retinal blood flow is mainly controlled through autoregulation by both myogenic and local metabolic mechanisms. However, previously published data using laser Doppler velocimetry and fluorescein angiography to assess retinal blood flow revealed that retinal perfusion seems to be autoregulated until an acute IOP increase occurs above 30 mmHg [33,34,35]. These values are lower than the reported autoregulatory capacity for the blood flow around the optic nerve head (ONH). The perfusion of the ONH seems to be relatively stable up to IOP values of 40 mmHg or even higher [36,37]. Applying force to the globe through tight-squeeze blinking or OM can lead to an acute IOP increase from 18 to 56 mmHg [38]. Therefore, one can assume a failure of retinal blood flow autoregulation due to the acute increase of IOP during OM, followed by a lower IOP with dilatation of the retinal vessels. However, ONH perfusion stays steady through higher resistance against IOP changes.

In contrast to the retinal vascular network, choroidal vessels have a rich supply of autonomic vasoactive innervation with a poor autoregulatory capability [5,19]. Immediately after OM, SFCT was significantly thicker than before OM. The increase of choroidal thickness due to lower IOP is in line with data published by Zhang et al. who reported a choroidal thickness increase of 2.8 µm for every 1 mmHg decrease in IOP in glaucomatous eyes [39]. Beyond this, we suppose a fluid shift from the choroidal vessels into the interstitium due to the pressure of OM impacting the globe. Therefore, the SFCT in our study group demonstrated a higher increase than expected just from the IOP drop alone.

CC showed a significant higher perfusion after OM, correlating positively with SCPP, while SLP and HLP did not change. As pointed out previously, choroidal vessel perfusion is mostly determined by autonomic innervation, making systemic BP the main influencing factor on choroidal perfusion [40]. As OM should have a negligible influence on BP, choroidal perfusion changes were not expected after performing OM. However, we were able to demonstrate a significant increase of CCP after OM. Farjood et al. recently proved an increased expression of major angiogenic factors in primary porcine retinal pigment epithelium (RPE) cells due to mechanical stress [41]. Through the mechanical force of OM in the stretching of the RPE, the local level of the vascular endothelial growth factor could change, which might lead to alterations in choroidal permeability. Since the CC is located just beneath the RPE, the influence of local angiogenetic factors is the greatest of all the choroidal sublayers.

The present study has some limitations. Since there is currently no way of measuring IOP simultaneously with the OM, we do not know the maximum IOP induced by globe compression. Furthermore, participants performed OM by themselves after detailed instruction. Indeed, a standardized procedure cannot be guaranteed since we are unable to measure the applied force. To reduce this possible confounding factor, only participants with a medical background and who were familiar with OM were recruited. It should be noted that we only used the Otsu image processing algorithm and that another algorithm might have led to a divergent outcome, as it has been shown that algorithms do have different discriminatory abilities. Furthermore, the manual recording of SFCT may represent a potential bias. However, all manual measurements were performed by two experienced graders (F.R. and M.P.) and the average values were used for statistical analysis. Furthermore, we cannot suggest global impairment of the choroid from only evaluating SFCT. A wider range of the choroidal examination area, or rather measuring the total choroidal volume, may be more conclusive. As a simple correlation analysis was used, we may have missed out on physiological interactions between the examined tissues. Finally, the relatively small sample size may represent a limiting factor leading to a mainly exploratory data analysis. Nevertheless, strong significant diurnal variations were found. To corroborate our findings, further studies with a larger number of participants and especially with an older study cohort will be necessary, since the young healthy adults included here are not the typical risk group for retinal artery occlusions. Furthermore, future studies should focus on subanalysis of the different vessel types to investigate whether the larger vessels or small capillaries react to OM with perfusion changes.

## 5. Conclusions

In conclusion, this prospective observational study demonstrated a significant perfusion increase in both the retinal capillary plexus as well as the CC after performing OM in healthy eyes, presumed to be due to failure of autoregulatory mechanisms. These results may indicate a positive effect of OM as a treatment option in patients with acute retinal artery occlusion by improving the retinal perfusion. However, to confirm this hypothesis further OCTA studies should focus on OM in diseased patients as well as age-matched healthy controls.

## Figures and Tables

**Table 1:** Demographic and clinical data of enrolled participants.

Parameter	Median (Min; Max)
Age (years)	29 (24; 36)
Axial length (mm)	24.13 (23.05; 26.61)
BCVA (logMAR)	−0.1 (−0.1; 0.0)
MAP (mmHg)	94 (85.7; 99.3)

BCVA, best-corrected visual acuity; MAP, mean arterial pressure.

**Table 2:** Anatomical and functional parameters of included eyes before and after ocular massage.

Parameter	Baseline Median (min;max)	After Ocular Massage Median (min;max)	Wilcoxon Signed-Rank (*p*-Value)
IOP (mmHg)	16 (12;21)	14 (9;19)	<0.001
CMT (µm)	269 (233;318)	268 (236;320)	0.114
TMV (mm³)	10.3 (9.6;11.2)	10.3 (9.5;11.2)	0.323
SFCT (µm)	342.7 (194;447)	367 (187;513)	0.005
RPCP (%)	45.1 (41.6;47.4)	44.8 (41.5;46.9)	0.407
SCPP (%)	27.7 (24.2;32.8)	30.4 (26.5;37.9)	<0.001
DCPP (%)	38.6 (35.1;40.1)	38.7 (37.4;42.3)	0.004
CCP (%)	47.4 (42.5;52)	48.2 (45;53.9)	0.008
SLP (%)	59.4 (54.8;64.8)	59.4 (54.8;63)	0.304
HLP (%)	62.2 (58.5;84.6)	62 (57;85.3)	0.554

IOP, intraocular pressure; CMT, central macular thickness; TMV, total macular volume; SFCT, subfoveal choroidal thickness; RPCP, radial peripapillary capillary perfusion; SCPP, superficial capillary plexus perfusion; DCPP, deep capillary plexus perfusion; CCP, choriocapillaris perfusion; SLP, Sattler’s layer perfusion; HLP, Haller’s layer perfusion.

**Table 3:** Correlation analysis of changes in intraocular pressure and perfusion values due to ocular massage.

Parameter		IOP	RPCP	SCPP	DCPP	CCP	SLP	HLP
**IOP**	CC	1	−0.135	−0.318	0.046	−0.163	0.082	−0.076
	p		0.559	0.160	0.843	0.481	0.723	0.742
**RPCP**	CC	−0.135	1	0.096	0.307	−0.029	−0.315	−0.123
	p	0.559		0.68	0.176	0.901	0.164	0.594
**SCPP**	CC	−0.318	0.096	1	0.354	0.704	−0.518	−0.455
	p	0.160	0.68		0.115	<0.001	0.016	0.09
**DCPP**	CC	0.046	0.307	0.354	1	0.369	−0.38	−0.315
	p	0.843	0.176	0.115		0.1	0.089	0.17
**CCP**	CC	−0.163	−0.029	0.704	0.369	1	−0.373	−0.56
	p	0.481	0.901	<0.001	0.1		0.095	0.008
**SLP**	CC	0.082	−0.315	−0.518	−0.38	−0.373	1	0.703
	p	0.723	0.164	0.016	0.089	0.095		<0.001
**HLP**	CC	−0.076	−0.123	−0.455	−0.315	−0.56	0.703	1
	p	0.742	0.694	0.09	0.17	0.008	<0.001	

CC, correlation coefficient; *p*, *p*-value.

## References

[1] Robinson F., Riva C.E., Grunwald J.E., Petrig B.L., Sinclair S.H. (1986). Retinal blood flow autoregulation in response to an acute increase in blood pressure. Investig. Ophthalmol. Vis. Sci..

[2] Dumskyj M.J., Eriksen J.E., Doré C.J., Kohner E.M. (1996). Autoregulation in the human retinal circulation: Assessment using isometric exercise, laser Doppler velocimetry, and computer-assisted image analysis. Microvasc. Res..

[3] Siegfried F., Rommel F., Rothe M., Brinkmann M.P., Sochurek J.A.M., Freitag J., Grisanti S., Ranjbar M. (2019). Evaluating diurnal changes in choroidal sublayer perfusion using optical coherence tomography angiography. Acta Ophthalmol..

[4] Rommel F., Rothe M., Kurz M., Prasuhn M., Grisanti S., Ranjbar M. (2020). Evaluating diurnal variations in retinal perfusion using optical coherence tomography angiography. Int. J. Retina Vitr..

[5] Polska E., Simader C., Weigert G., Doelemeyer A., Kolodjaschna J., Scharmann O., Schmetterer L. (2007). Regulation of choroidal blood flow during combined changes in intraocular pressure and arterial blood pressure. Investig. Ophthalmol. Vis. Sci..

[6] Luo X., Shen Y., Jiang M., Lou X., Shen Y. Ocular Blood Flow Autoregulation Mechanisms and Methods. https://www.hindawi.com/journals/joph/2015/864871/.

[7] Guidoboni G., Harris A., Cassani S., Arciero J., Siesky B., Amireskandari A., Tobe L., Egan P., Januleviciene I., Park J. (2014). Intraocular pressure, blood pressure, and retinal blood flow autoregulation: A Mathematical model to clarify their relationship and clinical relevance. Investig. Ophthalmol. Vis. Sci..

[8] Korenfeld M.S., Dueker D.K. (2016). Review of external ocular compression: Clinical applications of the ocular pressure estimator. Clin. Ophthalmol..

[9] Turner D.C., Girkin C.A., Downs J.C. (2019). The magnitude of intraocular pressure elevation associated with eye rubbing. Ophthalmology.

[10] Huber-van der Velden K.K., Lux A., Severing K., Klamann M.K.J., Winterhalter S., Remky A. (2012). Retrobulbar hemodynamics before and after oculopression with and without dorzolamide. Curr. Eye Res..

[11] Ffytche T.J. (1974). A rationalization of treatment of central retinal artery occlusion. Trans. Ophthalmol. Soc. UK.

[12] Beatty S., Au Eong K.G. (2000). Acute occlusion of the retinal arteries: Current concepts and recent advances in diagnosis and management. J. Accid. Emerg. Med..

[13] Cugati S., Varma D.D., Chen C.S., Lee A.W. (2013). Treatment options for central retinal artery occlusion. Curr. Treat. Options Neurol..

[14] Schrag M., Youn T., Schindler J., Kirshner H., Greer D. (2015). Intravenous fibrinolytic therapy in central retinal Artery occlusion: A patient-level meta-analysis. JAMA Neurol..

[15] Rommel F., Siegfried F., Kurz M., Brinkmann M.P., Rothe M., Rudolf M., Grisanti S., Ranjbar M. (2018). Impact of correct anatomical slab segmentation on foveal avascular zone measurements by optical coherence tomography angiography in healthy adults. J. Curr. Ophthalmol..

[16] Spaide R.F., Fujimoto J.G., Waheed N.K., Sadda S.R., Staurenghi G. (2018). Optical coherence tomography angiography. Prog. Retin. Eye Res..

[17] Rommel F., Brinkmann M.P., Sochurek J.A.M., Prasuhn M., Grisanti S., Ranjbar M. (2020). Ocular blood flow changes impact visual acuity gain after surgical treatment for idiopathic epiretinal membrane. J. Clin. Med..

[18] Lauermann J.L., Woetzel A.K., Treder M., Alnawaiseh M., Clemens C.R., Eter N., Alten F. (2018). Prevalences of segmentation errors and motion artifacts in OCT-angiography differ among retinal diseases. Graefes Arch. Clin. Exp. Ophthalmol. Albrecht Von Graefes Arch. Klin. Exp. Ophthalmol..

[19] Rommel F., Siegfried F., Sochurek J.A.M., Rothe M., Brinkmann M.P., Kurz M., Prasuhn M., Grisanti S., Ranjbar M. (2019). Mapping diurnal variations in choroidal sublayer perfusion in patients with idiopathic epiretinal membrane: An optical coherence tomography angiography study. Int. J. Retina Vitr..

[20] Gabriel M., Esmaeelpour M., Shams-Mafi F., Hermann B., Zabihian B., Drexler W., Binder S., Ansari-Shahrezaei S. (2017). Mapping diurnal changes in choroidal, Haller’s and Sattler’s layer thickness using 3-dimensional 1060-nm optical coherence tomography. Graefes Arch. Clin. Exp. Ophthalmol. Albrecht Von Graefes Arch. Klin. Exp. Ophthalmol..

[21] Esmaeelpour M., Kajic V., Zabihian B., Othara R., Ansari-Shahrezaei S., Kellner L., Krebs I., Nemetz S., Kraus M.F., Hornegger J. (2014). Choroidal Haller’s and Sattler’s layer thickness measurement using 3-dimensional 1060-nm optical coherence tomography. PLoS ONE.

[22] Otsu N. (1979). A threshold selection method from gray-level histograms. IEEE Trans. Syst. Man Cybern..

[23] Nicolò M., Rosa R., Musetti D., Musolino M., Saccheggiani M., Traverso C.E. (2017). Choroidal vascular flow area in central serous chorioretinopathy using swept-source optical coherence tomography angiography. Investig. Ophthalmol. Vis. Sci..

[24] Rothe M., Rommel F., Klapa S., Humrich J.Y., Nieberding R., Lange T., Sochurek J.A.M., Plöttner P., Grisanti S., Riemekasten G. (2019). Evaluation of retinal microvascular perfusion in systemic sclerosis: A case-control study. Ann. Rheum. Dis..

[25] Ali M., Akhtar F. (2011). Ocular digital massage for the management of post-trabeculectomy underfiltering blebs. J. Coll. Physicians Surg.Pak..

[26] García-Medina J.J., del-Río-Vellosillo M., Rubio-Velázquez E., López-Bernal M.D., Zafra-Pérez J.J. (2019). Focal vitreomacular traction: Resolution after ocular massage. Am. J. Ophthalmol. Case Rep..

[27] Mansour A.M., Haddad R.S. (2002). Corneal topography after ocular rubbing. Cornea.

[28] Lam A.K.C., Chen D. (2007). Effect of ocular massage on intraocular pressure and corneal biomechanics. Eye.

[29] Robbins R., Blumenthal M., Galin M.A. (1970). Reduction of vitreous weight by ocular massage. Am. J. Ophthalmol..

[30] Luo X., Shen Y.-M., Jiang M.-N., Lou X.-F., Shen Y. (2015). Ocular blood flow autoregulation mechanisms and methods. J. Ophthalmol..

[31] Puchner S., Schmidl D., Ginner L., Augustin M., Leitgeb R., Szegedi S., Stjepanek K., Hommer N., Kallab M., Werkmeister R.M. (2020). Changes in retinal blood flow in response to an experimental increase in IOP in healthy participants as assessed with doppler optical coherence tomography. Investig. Ophthalmol. Vis. Sci..

[32] Laties A.M. (1967). Central retinal artery innervation. Absence of adrenergic innervation to the intraocular branches. Arch. Ophthalmol. Chic. Ill. 1960.

[33] Riva C.E., Sinclair S.H., Grunwald J.E. (1981). Autoregulation of retinal circulation in response to decrease of perfusion pressure. Invest. Ophthalmol. Vis. Sci..

[34] Schulte K., Wolf S., Arend O., Harris A., Henle C., Reim M. (1996). Retinal hemodynamics during increased intraocular pressure. Ger. J. Ophthalmol..

[35] Zhang Q., Jonas J.B., Wang Q., Chan S.Y., Xu L., Wei W.B., Wang Y.X. (2018). Optical coherence tomography angiography vessel density changes after acute intraocular pressure elevation. Sci. Rep..

[36] Riva C.E., Hero M., Titze P., Petrig B. (1997). Autoregulation of human optic nerve head blood flow in response to acute changes in ocular perfusion pressure. Graefes Arch. Clin. Exp. Ophthalmol. Albrecht Von Graefes Arch. Klin. Exp. Ophthalmol..

[37] Pillunat L.E., Anderson D.R., Knighton R.W., Joos K.M., Feuer W.J. (1997). Autoregulation of human optic nerve head circulation in response to increased intraocular pressure. Exp. Eye Res..

[38] McMonnies C.W. (2007). Abnormal rubbing and keratectasia. Eye Contact Lens.

[39] Zhang X., Cole E., Pillar A., Lane M., Waheed N., Adhi M., Magder L., Quigley H., Saeedi O. (2017). The effect of change in intraocular pressure on choroidal structure in glaucomatous Eyes. Investig. Ophthalmol. Vis. Sci..

[40] Delaey C., Van De Voorde J. (2000). Regulatory mechanisms in the retinal and choroidal circulation. Ophthalmic Res..

[41] Farjood F., Ahmadpour A., Ostvar S., Vargis E. (2020). Acute mechanical stress in primary porcine RPE cells induces angiogenic factor expression and in vitro angiogenesis. J. Biol. Eng..

